# Alternative prosthodontic‐based treatment of a patient with hypocalcified type Amelogenesis Imperfecta

**DOI:** 10.1002/ccr3.1005

**Published:** 2017-05-23

**Authors:** Anca Jivanescu, Antonio Miglionico, Souman Barua, Simona Ioana Hategan

**Affiliations:** ^1^ Department of ProsthodonticsFaculty of Dentistry Timisoara“Victor Babes” University of Medicine and Pharmacy TimisoaraTimisoaraRomania; ^2^ Faculty of Dentistry Timisoara“Victor Babes” University of Medicine and Pharmacy TimisoaraTimisoaraRomania

**Keywords:** Adhesive cementation, Amelogenesis Imperfecta, zirconia fixed partial denture

## Abstract

The Amelogenesis Imperfecta is associated with malocclusion and usually requires an interdisciplinary treatment. Due to the patient's refusal of orthodontic treatment, prosthodontics‐based treatments alternative was considered and planned. The patient was treated with zirconia‐based fixed partial dentures, which resulted in improved occlusion, better oral health, and improved esthetic appearance.

## Introduction

Amelogenesis Imperfecta is a hereditary condition affecting the deciduous and permanent dentition. There are several forms of AI. These forms have differences in the enamel development stages and the enamels’ appearance and include: Amelogenesis Imperfecta Hypoplastic (Type I), Amelogenesis Imperfecta Hypomaturation (Type II), Amelogenesis Imperfecta Hypocalcified (type III), and Hypomaturation–hypoplastic with Taurodontism 1, 2.

Amelogenesis Imperfecta Hypoplastic (Type I) is characterized by highly reduced enamel matrix layer due to lack of ameloblast cells during the formation 1, 2. Amelogenesis Imperfecta Hypomatured (Type II) occurs due to interruption of the enamel maturation process resulting in normal enamel thickness, but with no hardness or translucency 2.

Amelogenesis Imperfecta Hypocalcified or hypomineralised (Type III) is characterized by softer enamel with irregular matrix formation. The erupted teeth have initially normal appearance, but the dentin becomes exposed, and the enamel color progresses from light yellow‐brown to orange, and ultimately the color becomes deep brown to black, due to food stains 1, 2.

Achieving functional and esthetic improvement for teeth affected by Amelogenesis Imperfecta is a challenging process. Treatment options and planning usually require complex therapeutic approaches by a multidisciplinary team, and depend on the severity of the intraoral condition and the socioeconomic background. Prosthodontic treatment plays a key role, and effective communications among different specialities, prosthodontics, endodontics, and periodontics are needed 3, 4, 5, 6, 7.

Herein we present a case of prosthodontics‐based esthetic and functional rehabilitation in a patient with Amelogenesis Imperfecta and provide a literature review on therapeutic approaches.

## Clinical Report

A 17‐year‐old male patient with Amelogenesis Imperfecta was referred to the Department of Prosthodontics, Faculty of Dentistry, Victor Babes University, Timisoara, Romania due to chief complaint of highly sensitive teeth with gross attrition.

Initial medical, dental, and social histories were obtained and the clinical examination revealed that the enamel layer was thin, the dentin was exposed in many teeth, and the enamel color was light yellow‐brown to orange. It was determined that the patient was affected by hypocalcified (Type III) Amelogenesis Imperfecta. The patient's choice was to restore function and esthetics, without orthodontic treatment.

**Figure 1:**
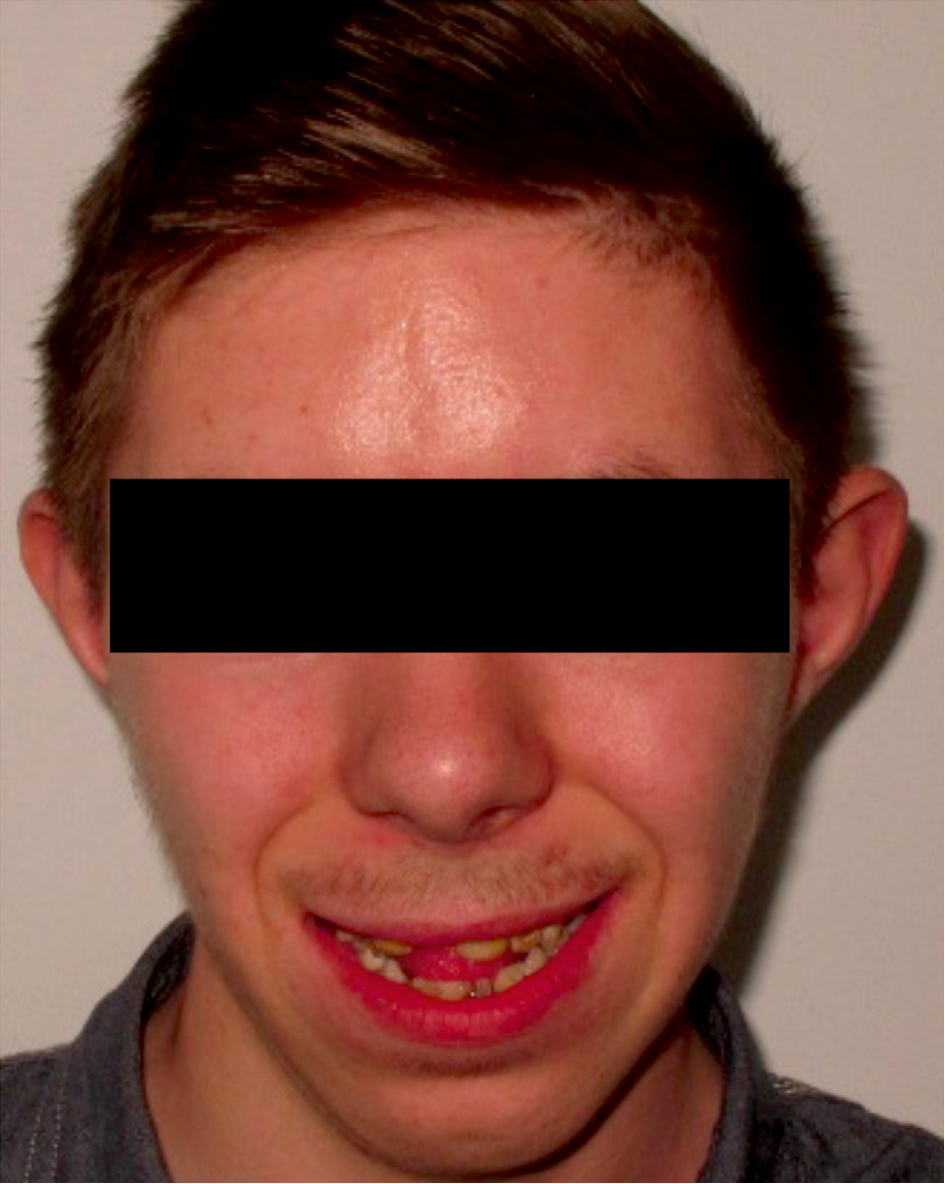
Full face photographs of the patient with AI at the presentation.

Extraoral examination and facial analysis showed a facial asymmetry with a mandibular prognathism and cross bite (Fig. 1). The patient explained that his socializing activities were limited due to his appearance.

**Figure 2:**
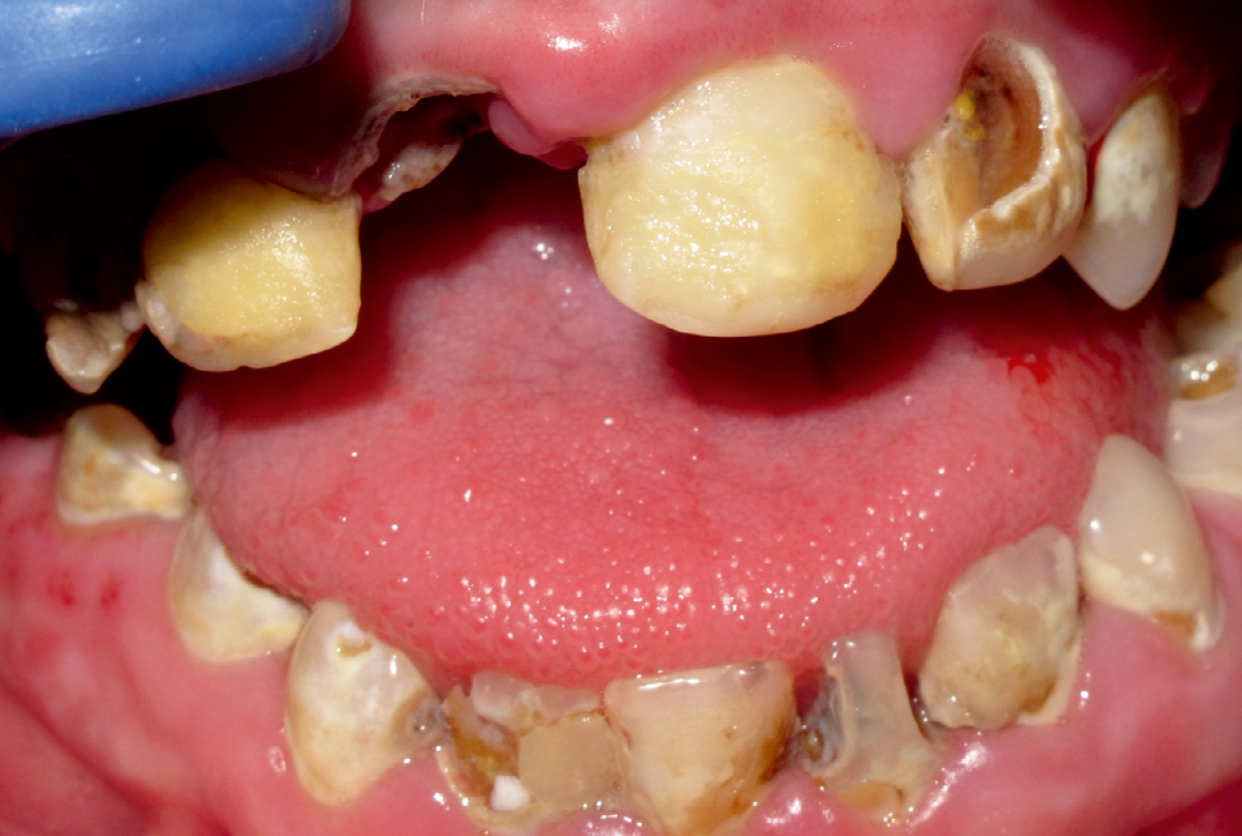
Aspect of the hypocalcification of the enamel and the exposed dentin and decayed dentin.

Oral hygiene was poor, with significant plaque accumulations. Hypocalcification in enamel was clearly visible and dentin was exposed (Fig. 2).

**Figure 3:**
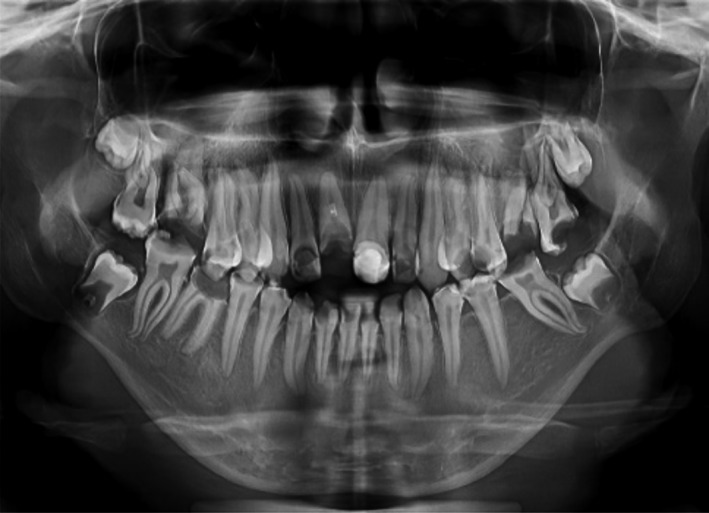
Orthopantomography of the patient.

Radiographic examination (Fig. 3) revealed:

1. multiple untreated simple and complex carious lesions;
2. necrotic pulp in some teeth (# 6, 9, 10, 12, 13, 18, 23, 24, 25,26, 28, and 29);
3. cross‐bite occlusion of Kennedy class III with two modifications in maxilla and Class III Kennedy with one modification in mandible;
4. gingivitis and localized gingival hyperplasia.

The treatment’ goals were to develop an overall oral health improvement, and to restore function with an esthetic appearance.

The standard treatment options requiring interdisciplinary approaches were discussed with the patient. Due to the patient's refusal of the orthodontics therapy, a decision was made to pursue with mainly a prosthodontics approach, preceded by the following preprosthodontic procedures:

1. Hygiene maintenance until repeated visits confirmed compliance.
2. Extraction of teeth deemed nonrestorable # 7, 8, 14, 15, 30.
3. Endodontic treatment of teeth # 6, 9, 10, 12, 13, 18, 23, 24, 25, 26, 28, 2.
4. Crown lengthening of teeth # 4, 5, 6, 12, 13, 20, 21, 28, 29.
5. Occlusal splint to be worn for 2 months prior to initiating prosthetic treatment.

**Figure 4:**
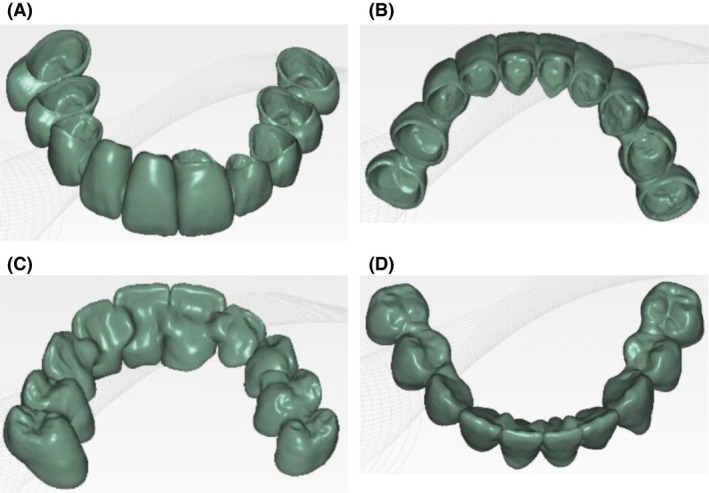
Digital design of provisional restorations.

**Figure 5:**
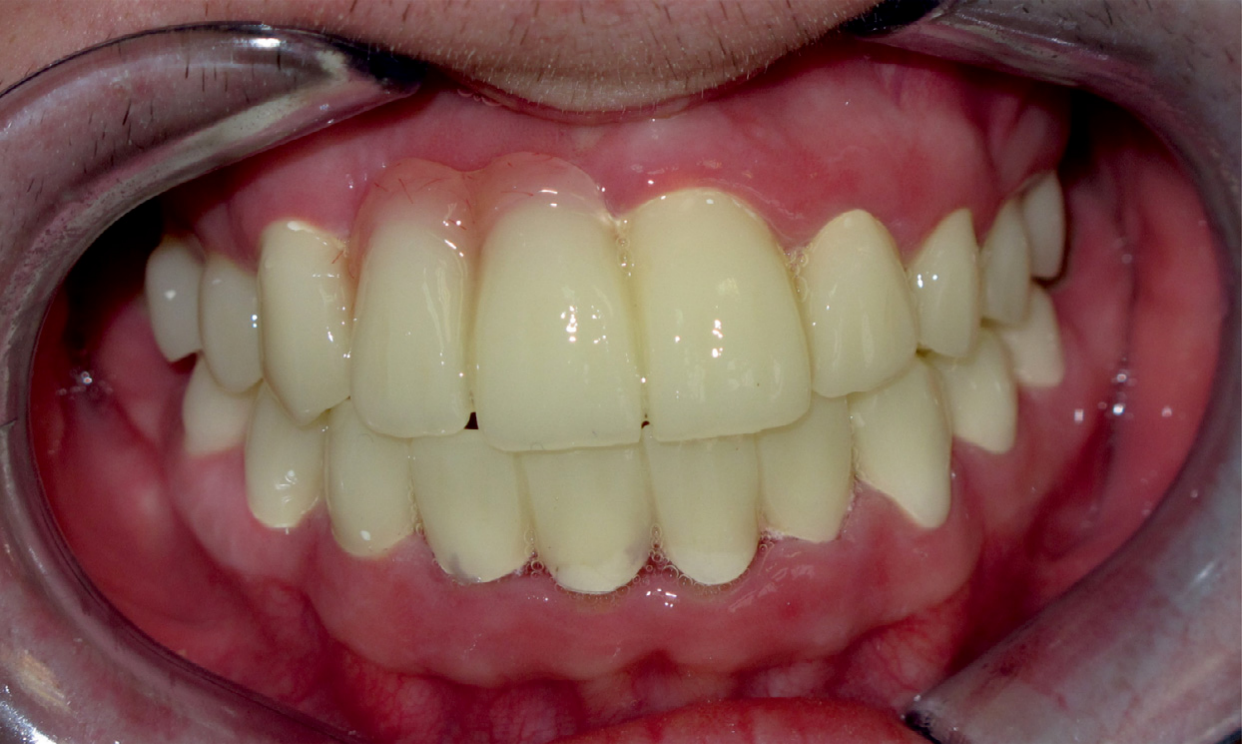
Provisional restorations after cementation.

Next, the treatment's potential outcome was assessed by a diagnostic wax‐up. In order to obtain the appropriate vertical dimension of occlusion in harmony with facial esthetics, two sets of provisional restorations were fabricated. The first provisional restorations were made by a direct technique based on the preliminary wax‐up model. After completing the preparations, models were scanned and a digital design of the future prosthetic restoration was proposed (Fig. 4A–D). Based on this design, the second provisional restorations were milled from resin blocks (Telio CAD; Ivoclar Vivadent, Schaan, Lichtenstein) and cemented with temporary cement (RelyX NE; 3MESPE Seefeld, Germany) (Fig. 5).

After the trial period with provisional restorations, the definitive impression was taken in two steps technique using addition silicone (Elite HD; Zhermack, Badia Polesine, Italy), and jaw relation record transferred to an articulator (Artex; Armann Girbach, Koblach, Austria).

All maxillary and mandibular restorations were fabricated with zirconia‐based crowns. Restorations included:

1. single crown restoration: # 4, 5, 12, 13, 20, 21, 28, 29;
2. fixed dental prosthesis: # 6 to 11 and #23 to 26.

**Figure 6:**
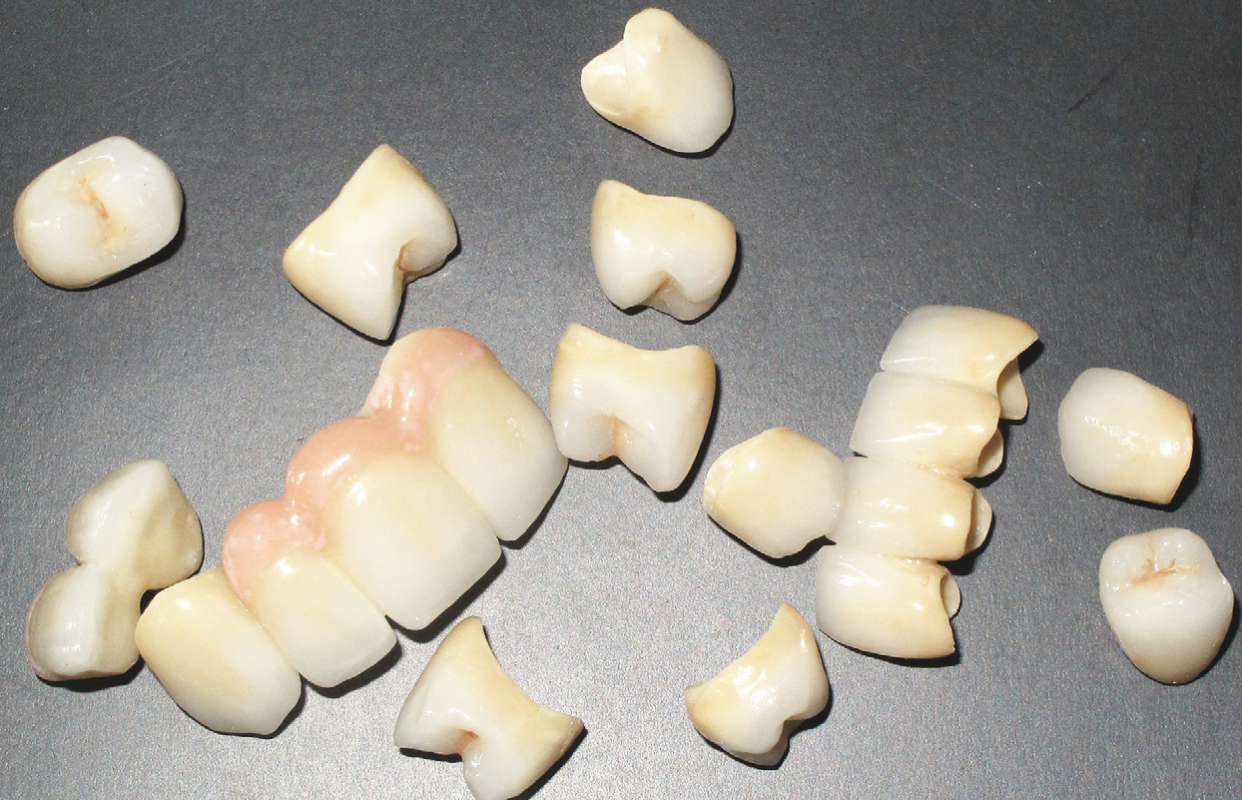
Lithium disilicate veneered zirconia fixed dental prostheses.

Lithium disilicate(e.max Ceram; Ivoclar Vivadent, GmbH,Tech Gate Vienna, Donau‐City‐Straße 1,1220 Viena, Austria) was used to veneer the zirconia framework, along with pink ceramic to restore the soft tissue contours at the incisors area (Fig. 6).

The cementation protocol follows several steps. The intaglio surface of the restorations was air‐particle abraded with 50 μm aluminum oxide particles at a distance of 1–2 cm.

After trying the prosthetic restoration in patient's mouth, Ivoclean (Ivoclar/Vivadent)) was used for 1 min and later washed with water. Then one layer of a primer (Z Prime Plus; BISCO, Schaumburg, Illinois, USA) was applied in the inner surface of zirconia crowns, left for 1 min and dried with air. The abutments were cleaned with fluoride‐free cleaning paste without polishing, than washed and dried. A self‐adhesive resin cement Theracem (BISCO, Schaumburg, Illinois, USA) was applied and the restorations seated and light cured for about 3‐sec buccal and 3‐sec palatal. Excess cement was removed with a curette. Another 60 sec of curing light was used for finalizing the cementation.

**Figure 7:**
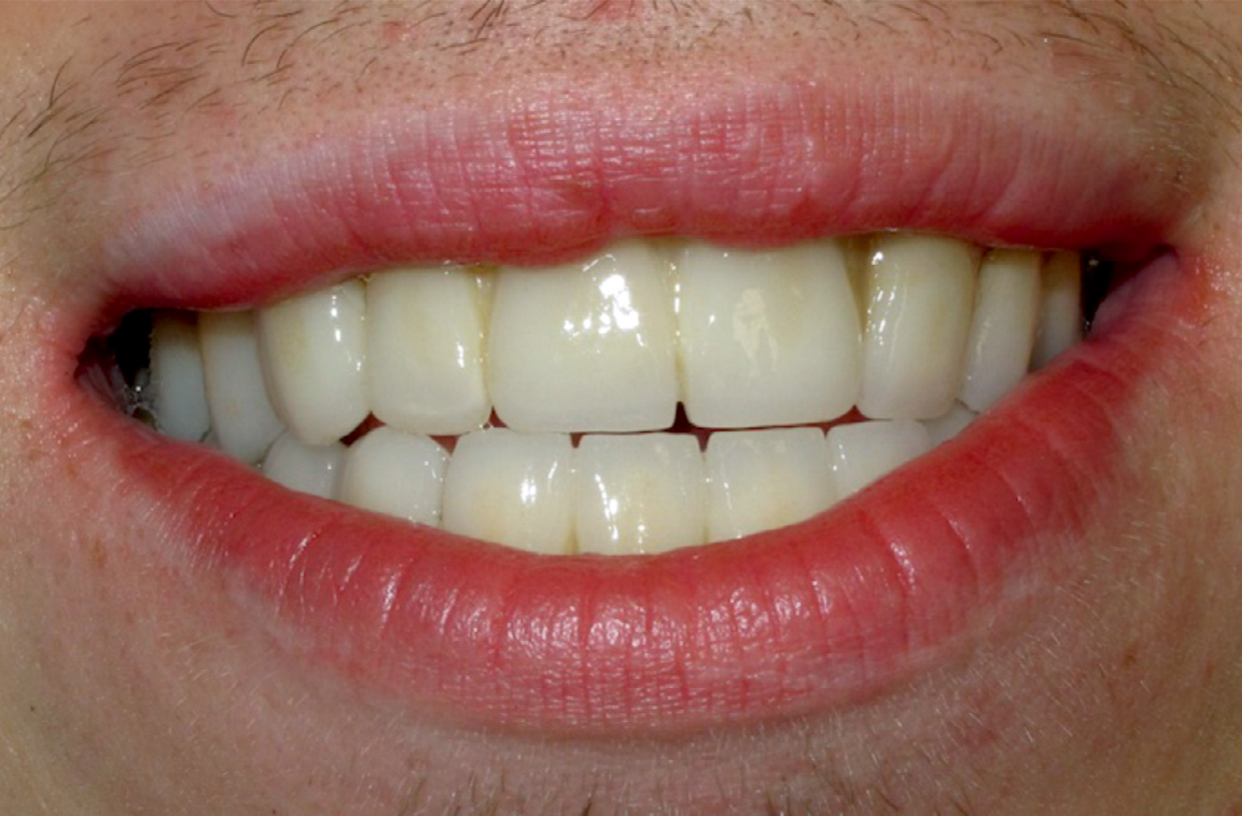
Final smile of the patient 2 weeks after final cementation of zirconia fixed dental prostheses.

Initial posttreatment follow‐up at 2 weeks demonstrated satisfactory oral health (Fig. 7). Six‐month and 12‐month follow‐up showed persistent beneficial outcome, with no degeneration of the restoration.

## Discussion

Amelogenesis Imperfecta is a rare disorder with a challenging management, especially in later stages. An interdisciplinary approach including orthodontic treatment procedures is the accepted gold standard to ensure optimal outcomes including oral health and function, as well as improved esthetics and function in cases of Amelogenesis Imperfecta 8, 9, 10, 11, 12.

More recently, it was suggested that less invasive treatment alternative with direct composite resin restorations, especially in cases of Amelogenesis Imperfecta without malocclusion, may also be employed 13, 14.

In this case of Amelogenesis Imperfecta type III, we demonstrate that a therapeutic strategy of zirconia‐based prosthetic restorations alone successfully improved oral function and oral health, and restored the esthetic appearance. We also present evidence of persistent benefit to up to 12 month of follow‐up. The bonding protocol of zirconia‐based restoration followed a precise sequence of air‐borne particle abrasion, zirconia primer and resin cement, in order to improve the longevity of the adhesion 15.

Thus, an alternative prosthodontics‐only based treatment may offer less invasive treatment options.

However, randomized controlled trials with adequate sample size and longer follow‐up are required to establish the validity of this treatment approach and to assess persistent benefits on functional and esthetic outcomes in these cases.

## Conclusion

The described prosthodontics strategy with zirconia‐based restoration and well‐adjusted vertical dimension of occlusion, resulted in the recovery of the masticatory function, improved oral health, and esthetic appearance in this patient, who was eventually able to resume his social life. This suggests that careful planning and patient‐specific alternative prosthodontics‐based treatment may be successful when multidisciplinary approaches are not an option.

## Conflict of Interest

The materials used in this case report are mentioned for clinical study purpose only. The authors do not have any financial interest in the companies whose materials are included in this article.

## Authorship

AJ: coordinated the treatment, summarized case information, and drafted the manuscript. AM: carried out the treatment, participated in the follow‐up of the patient. SB: participated in drafting the manuscript. SIH: participated in patient care. All authors read and approved the final manuscript.
